# Latent Health Risk Classes Associated with Poor Physical and Mental Outcomes in Workers with COPD from Central Appalachian U.S. States

**DOI:** 10.3390/ijerph17186798

**Published:** 2020-09-17

**Authors:** Michael Stellefson, Min Qi Wang, Jo Anne G. Balanay, Rui Wu, Samantha R. Paige

**Affiliations:** 1 Department of Health Science, The University of Alabama, Tuscaloosa, AL 35487, USA; 2 Department of Behavioral and Community Health, University of Maryland, College Park, MD 20742, USA; mqw@umd.edu; 3 Department of Health Education and Promotion, East Carolina University, Greenville, NC 27858, USA; balanayj@ecu.edu; 4 Department of Computer Science, East Carolina University, Greenville, NC 27858, USA; wur18@ecu.edu; 5 STEM Translational Communication Center, University of Florida, Gainesville, FL 32611, USA; paigesr190@ufl.edu

**Keywords:** COPD, rural health, Appalachia, health-related quality of life

## Abstract

Adults who work in the Central Appalachian region of the United States (U.S.) are disproportionately affected by Chronic Obstructive Pulmonary Disease (COPD). While there is a socio-demographic profile of adults with COPD who are at increased risk for physical and mental distress, the risk factors that uniquely affect the health-related quality of life (HRQoL) of Central Appalachian workers with COPD are unknown. Therefore, we conducted a latent class analysis of 2016 and 2017 Behavioral Risk Factor Surveillance System data from 1326 currently employed adults with COPD living in four U.S. states (KY, NC, TN, and WV) within the Central Appalachian Region. Drawing from the social ecological model, we identified associations between theoretically informed risk indicators—comorbid health conditions, substance use and abuse, and limited access to healthcare—on three HRQoL variables, including infrequent (0–13 days) or frequent (≥14 days) physical distress, mental distress, and limited activity due to poor health over the past 30 days. Workers at high risk for comorbid conditions reported more frequent physical distress, mental distress, and activity limitations as compared to those at low risk. Workers reporting difficulty accessing healthcare were no more likely to report physical or mental distress when compared to workers with adequate access to healthcare; however, those with limited healthcare access did report more frequent activity limitation due to poor health. Interestingly, workers with COPD at high risk for substance use and abuse were no more likely to report poor HRQoL outcomes compared to those at low risk. Our findings have important implications for addressing indicators of poor health among Central Appalachian workers with COPD, especially those living with multiple comorbidities.

## 1. Introduction

Chronic Obstructive Pulmonary Disease (COPD) is a progressive respiratory condition that causes dyspnea (i.e., shortness of breath) which increases risks for physical immobility and emotional distress [1,2,3,4]. The highest quartile of COPD prevalence in the United States (U.S.) is concentrated in Central Appalachia, comprising the following states: Kentucky, Tennessee, West Virginia, and North Carolina. Central Appalachia represents one of the most disadvantaged regions in the country with states that have higher rural populations than the rest of the U.S. [5,6,7]. Adults residing in rural regions of the nation are at a higher risk for COPD than their non-rural counterparts [8]. Almost 40% of Appalachian counties have COPD mortality rates in the worst-performing national quintile [9,10]. Excessive COPD-related hospitalizations are also concentrated in Central Appalachia [9,11]. Empirical attention directed towards the determinants of COPD outcomes is imperative in this region.

Occupational COPD has been linked with exposure to non-specific vapors, gases, dust, and fumes, which may be attributed to factory and coal mining work that is common in the region [12,13,14,15,16,17]. The effect of occupational COPD on rural adults’ health outcomes does not occur in isolation [14]; rather, several system-level factors contribute to rural health disparities in COPD. For example, rural adults report a disproportionate exposure to environmental tobacco smoke [18,19], multiple co-morbidities that add a layer of complexity to planning and carrying out self-management programs [20], as well as a shortage of primary care providers [21]. Therefore, reliance on only individual-level factors that influence behavior change is unlikely to have potent effects on HRQoL in adults with chronic disease.

The social ecological model (SEM) is a health promotion framework widely employed in public health research to understand complex, multi-level factors that impact health outcomes [22]. According to the SEM, social and environmental systems in which individuals are embedded influence resource availability and behavioral tendencies and, in turn, the health status and disease prevalence within communities. SEM purports that health-enhancing structural changes are best fostered through understanding individual- and health care system-level correlates of HRQoL [23]. HRQoL measures dysfunction and disability related to chronic diseases at various ecological (e.g., individual, community) levels [24]. Despite the high prevalence of COPD in the Central Appalachian region, there has been relatively little done to identify how these factors impact the HRQoL of adults with COPD who are currently employed and living in resource-limited geographical areas such as Central Appalachia [25,26].

One recent secondary analysis of the 2016 Behavioral Risk Factor Surveillance System (BRFSS) dataset identified subgroups of U.S adults with COPD at the highest risk for physical and mental distress [27]. Most U.S. adults with COPD experienced physical (53.76%) and/or mental (58.23%) distress, with more frequent distress common among white females in the 45–64-year age group who were also identified as having lower socioeconomic status (SES). Respondents with COPD who reported intermediate to high risk behaviors (e.g., tobacco use and alcohol consumption) and intermediate to low use of preventive vaccinations (e.g., influenza and pneumonia) were more likely to report frequent distress compared to the low-risk respondents. This study provides important insight to the risk factors associated with mental and physical distress in COPD; however, there was little focus on the geographic residence of these adults and their employment status.

The employment status of workers living with COPD has been overlooked in areas where many COPD-related disparities exist [2,28]. Without this knowledge, researchers and practitioners are unable to identify and fully characterize how the health of workers with COPD may be affected by multi-level risk factors. Geographic Information System (GIS) mapping is a visual communication tool that is used to effectively depict multiple regional determinants of a health condition. These maps are especially useful in communicating about health risks to diverse stakeholders to increase risk awareness and promote risk mitigation [29]. As such, there is a clear and important need for population-based research identifying risk factors that affect workers with COPD in this disadvantaged U.S. region.

The purpose of this study was to analyze BRFSS data collected from employed respondents with COPD living in Central Appalachia to determine variability in HRQoL based on social ecological risk factors. The primary aim of this study was to: (1) classify social ecological (multi-level) health risks for employed workers with COPD who reside in four Central Appalachian U.S. states (Kentucky, Tennessee, West Virginia, and North Carolina); and (2) Identify subgroups of Central Appalachian workers with COPD who are at the highest risk for poor health and activity limitation due to multi-level risk factors. A secondary aim was to provide a visual representation of the findings via a risk mapping procedure. By using GIS-mapping procedures, we diagnosed and mapped individual and health system risk factors at the state level that have the largest effects on HRQoL among Central Appalachian workers with COPD. This study has important implications for addressing Goal 3 of the COPD National Action Plan [21], which is to, “collect, analyze, report, and disseminate COPD-related public health data that drive change and track progress.”

## 2. Materials and Methods

Administered by the Centers for Disease Control and Prevention (CDC) in all 50 states and 4 U.S. territories, the BRFSS is a population-based, random-digit-dialed telephone survey that monitors access to health care, certain health conditions, and behavioral health risks that contribute to the leading causes of disease and death among adults aged 18 years or older. The BRFSS empirically monitors numerous correlated and interdependent domains associated with HRQoL, including social ecological [22,30] risk factors at multiple levels, such as smoking (individual), employment status (occupational), and limited healthcare access (community). The U.S. States of Kentucky, North Carolina, Tennessee, and West Virginia were selected for analysis as they are included within the Central Appalachian region. This analysis included all 2016 and 2017 BRFSS respondents (*N* = 1326) from these U.S. states who answered “yes” to both of the following questions: (1) “Has a doctor, nurse, or other health professional EVER told you that you have chronic obstructive pulmonary disease (COPD), emphysema or chronic bronchitis?” and (2) are you “employed for wages” or “self-employed”?

**Figure 1:**
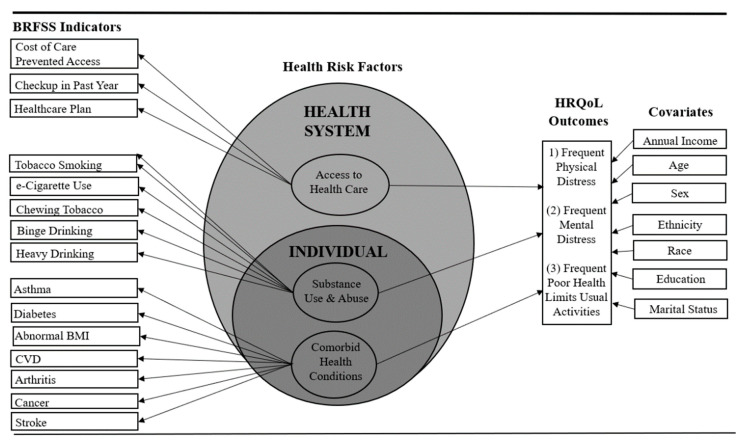
LCM approach to analyze 2016 and 2017 BRFSS data collected from employed adults with COPD living in KY, NC, TN, and WV (*N* = 1326). Fifteen indicators, three domains at two social ecological levels (i.e., individual, health system), three HRQoL outcomes, seven covariates. Data in this figure ARE adapted based on the indicators, domains, and covariates tested by Jiang and Zack [31] and Stellefson et al. [27].

BRFSS health risk indicators (*n* = 15) were categorized into three SEM domains—(1) comorbid health conditions (individual factor); (2) substance use and abuse (individual factor); and (3) limited access to healthcare (health system factor)—were selected as predictors of poor HRQoL outcomes in the Central Appalachian COPD worker population. Figure 1 demonstrates their theoretical relationship to one another.

Participants were asked if they have ever been told by a health professional that they had any of the following comorbid health conditions: asthma, diabetes, abnormal body mass index (BMI: underweight, overweight, or obese), cardiovascular disease (CVD), rheumatoid arthritis, cancer, and stroke. Participants were also asked about their history of substance use and abuse, including their: (1) current smoking status (every day or some days); (2) use of e-cigarettes or other electronic “vaping” products (yes or no); (3) use of chewing tobacco, snuff, or snus (every day or some days); (4) engagement in binge drinking (males having ≥5 drinks on one occasion in past 30 days or females having ≥4 drinks on one occasion in past 30 days); and (5) engagement in heavy drinking (men having >14 drinks per week and women having >7 drinks per week). Three responses indicated limited access to healthcare: (1) being uninsured (i.e., no coverage); (2) cost preventing access to care in the past year; and (3) no routine checkup in the past year.

Poor HRQoL was assessed for physical health (“Now thinking about your physical health, which includes physical illness and injury, for how many days during the past 30 days was your physical health not good?”), mental health (“Now thinking about your mental health, which includes stress, depression, and problems with emotions, for how many days during the past 30 days was your mental health not good?”), and limited usual activity (“During the past 30 days, for about how many days did poor physical or mental health keep you from doing your usual activities, such as self-care, work, or recreation?”). Quantifying these estimates in terms of days in the most recent month avoids the need to use more complex weights in aggregating and comparing data based on multiple choice questions [31,32]. Workers who experience mental or physical distress for at least 2 weeks (14 days) over the course of a month tend to function at lower levels [33]. Therefore, each of the dependent variables—physical health, mental health, and limited usual activities due to physical or mental health—were dichotomized in the following manner: 14 or more days (*frequent distress/limitation*), and 0 to 13 days (*infrequent distress/limitation*). Prior studies have coded BRFSS items similarly to identify distress impacting mental and physical HRQoL [27,34,35].

Multivariable analyses included age group (18–44, 45–64, ≥65), sex (male, female, refused), race (White, Black/African American, American Indian/Alaska Native, Asian, Pacific Islander, other, no preferred race), ethnicity (Hispanic, not Hispanic), annual income (USD <20,000, 20,000–34,999, 35,000–49,999, 50,000–74,999, AND ≥ 75,000), education level (never attended school or only attended kindergarten, grades 1–8, grades 9–11, grade 12 or General Education Degree (GED), college 1–3 years, college ≥ 4 years), and marital status (married, divorced, separated, widowed, never married, or member of an unmarried couple).

In this study, latent class modeling (LCM) was selected as the data analysis to evaluate how risk factors in the three broad social ecological domains (falling within the individual and health system levels of influence) were associated with BRFSS respondents’ HRQoL. LCM is an analysis that can be used to untangle complex relationships between multilevel health indicators to efficiently summarize predictors of physical and mental distress using underlying classes that define individual risk levels. The analysis collapses many discrete indicators into a few meaningful latent classes (i.e., categories that condense levels of a domain) and estimates the probability that a person belongs to a specific latent class. As in factor analysis, the LCM is used to classify cases according to their maximum likelihood class membership so individuals can be classified into mutually exclusive and exhaustive types, or latent classes, based on their pattern of answers on a set of variables so that the “high risk” to “low risk” groups can be identified. The response pattern is the unit of analysis [36]. LCM provides the prevalence of each latent class (marginal probabilities) and the class-specific response probabilities of each indicator (conditional probabilities) [37]. For example, a class 1 membership may detect that most respondents in class 1 show low presence of smoking, heavy drinking, or binge drinking, and we may classify this membership as “low risk” group; while a class 3 membership may detect that most respondents in class 3 show high presence of smoking, heavy drinking, or binge drinking, and we may classify this membership as “high risk” group. By the same token, we may find a class membership in between 1 and 3, and we may classify this membership as an “intermediate” risk group. To determine the most parsimonious model, the research team sequentially fit models from 1 to more latent classes [36], and subsequently compared successive models using the Bayesian Information Criterion (BIC). The model with the smallest BIC values was retained [38]. Models with three latent risk classes provided the best fit for health risk factor domains.

Multiple logistic regression was initially used to examine associations between demographic variables and the three measures of health-related distress. Subsequent logistic regression models entered three social ecological risk domains from the LCM analyses as the independent variables to examine associations with the three measures of health-related distress as the outcome variables (i.e., mental distress, physical distress, AND usual activity limitation due to poor health). Age, sex, race, ethnicity, education, annual income, and marital status were treated as covariates. The −2*log likelihood ratio, Pseudo-*R*^2^, odds ratios, and 95% CIs were used to evaluate the significance of the predictors and the model fit. All LCM analyses were conducted in Mplus (version 7.3) to accommodate the complex sampling design (i.e., strata, Primary Sampling Unit (PSU), and weight) of the BRFSS. SAS software (version 9.4, SAS Institute Inc., Cary, NC, USA) was used to conduct the logistic regression analyses.

To translate the findings of this study into a product that can be disseminated to public health practitioners and policy makers in Central Appalachia, we used geographic information system (GIS) mapping to visualize the prevalence of statistically significant state-level health risk factors in the Central Appalachian region. The integration of GIS and data visualization provides a more realistic presentation of spatial data. Specifically, GIS methods were used to visualize the prevalence of statistically significant risk factors that had the largest effect on HRQoL among workers with COPD in each Central Appalachian U.S. state. GIS provides functions that allow a user to examine the spatial relationships among entities. The visualization is to represent data in a way that may reveal patterns and relationships that are hard to detect using non-visual approaches, such as text and tables. Odds ratios of frequent physical distress, mental distress, and activity limitation were compared. Health risk indicators were visualized on a Google Map based on logistic regression results from Central Appalachian U.S. States (Kentucky, North Carolina, Tennessee, and West Virginia).

Colors were used to present the overall health risks of BRFSS respondents in each Central Appalachian U.S. state. These colors were expressed with three numbers: (1) hue, (2) saturation, and (3) value (HSV). The darker color means more serious health issues. For example, the HSV of red is (0 = Hue, 100 = Saturation, and 100 = Value). The (0,100,100) HSV is the most saturated (i.e., dark) red and thus represented the most serious health risks. In the Google Map, we used the average percentage of respondents reporting each health risk indicator to be the saturation value for each U.S. state. If the saturation was replaced with a smaller number, the color was lighter which meant the health risks were less critical based on our analysis.

## 3. Results

### 3.1. Demographic Characteristics

The demographics by physical distress, mental distress, and activity limitation are presented in Table 1 (*N* = 1326). Over half of the workers with COPD in Central Appalachian states were 45–64 years old and female. Almost 40% had graduated high school and more than half had at least some level of college education. Over 40% of respondents made less than USD 35,000 per year. Almost 50% of respondents were married, with the remainder identifying as being divorced, widowed, or separated. Around 20% of workers with COPD living in Central Appalachian U.S. states reported experiencing physical (20.97%) and/or mental (19.68%) distress over 14 days in the past month, indicative of “frequent distress.” Over 10% (11.16%) reported poor health leading to usual activity limitation.

### 3.2. Preliminary Logistic Regression Analyses

Workers with COPD in Central Appalachia who were younger were significantly (*p* < 0.01) more likely to report mental distress than workers with COPD over the age of 65 (18–44 years: OR = 3.58, 95% CI: 1.93–6.63; 45–64 years: OR = 1.97, 95% CI: 1.11–3.49). Female workers with COPD were also significantly (*p* < 0.01) more likely to report mental distress when compared to male workers with COPD (OR = 1.61, 95% CI: 1.17–2.23). African American workers with COPD were significantly (*p* < 0.05) less likely to report mental distress than Whites (OR = 0.44, 95% CI: 0.23–0.85). Workers with 4 years or more of college education were significantly (*p* < 0.05) less likely than workers without a high school diploma to report mental distress (OR = 0.48, 95% CI: 0.26–0.90).

Employed adults with COPD reporting incomes over USD 35,000 per year were significantly less likely to experience mental (*p* < 0.05; OR = 0.49–0.56) and physical distress (*p* < 0.01; OR = 0.41–0.58) as compared to those with lower incomes (USD <20,000 per year). Those with incomes over USD 50,000 per year (OR = 0.41–0.46) were also significantly (*p* < 0.01) less likely to report limited usual activities due to poor health as compared to those with lower incomes (USD <20,000 per year). These significant logistic regression results led the researchers to enter the covariates of age, sex, race, education, and annual income into subsequent LCM risk models examining associations with the three health-related distress outcomes (i.e., mental distress, physical distress, and usual activity limitation due to poor health). Ethnicity and marital status were also entered as covariates within risk models (see Figure 1).

### 3.3. Comorbid Health Condition Risk Classes

**Figure 2:**
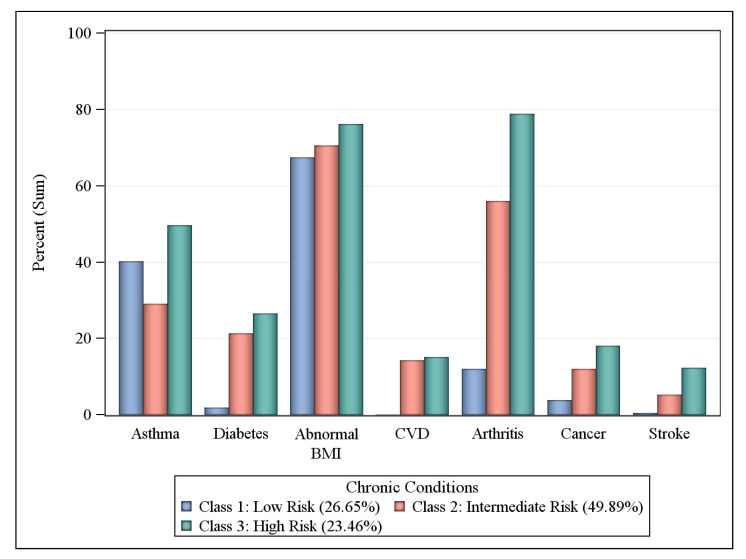
Probabilities of comorbid health condition by latent class, workers with COPD in KY, TN, NC, and WV BRFSS respondents, 2016 and 2017. Comorbid health conditions defined as having ever been told by a health professional that they had the condition of asthma, diabetes, cancer, CVD, arthritis, or stroke. Abnormal BMI defined as height and weight estimates that placed respondent as ‘underweight’, ‘overweight’, or ‘obese’.

About 23.46% of workers with COPD living in Central Appalachian U.S. states reported being in the high-risk comorbidity class for the seven health conditions (Figure 2). Over three-quarters of respondents in the high-risk class reported arthritis (79.57%), 72.54% had an abnormal BMI (i.e., were underweight, overweight, or obese), and close to one-half (53.79%) reported having asthma, and 28.20% reported diabetes. About 49.89% of workers with COPD belonged to an intermediate risk comorbidity class: 71.85% reported an abnormal BMI, 58.56% reported arthritis, 29.47% in this class reported having asthma, and 20.15% reported diabetes. In the remaining 26.65% at low risk for comorbidities, 65.88% reported an abnormal BMI and 40.63% reported having asthma.

### 3.4. Substance Use and Abuse Risk Classes

**Figure 3:**
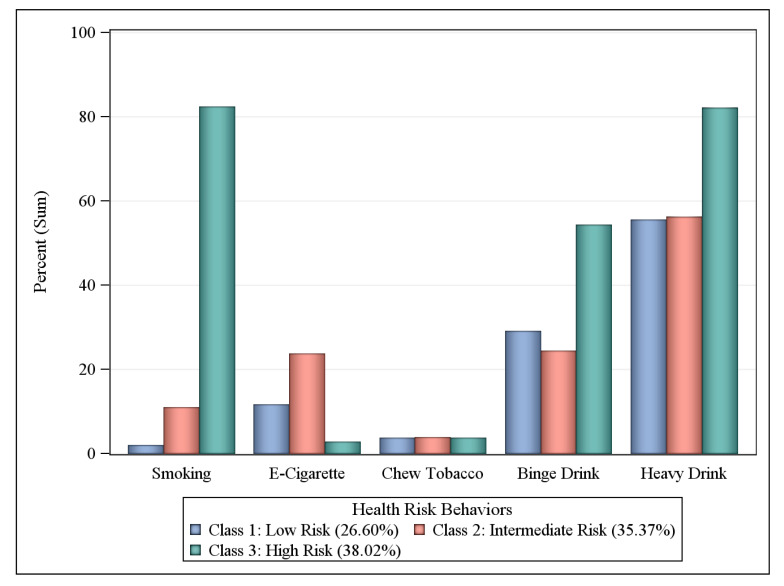
Prevalence probabilities of substance use and abuse behaviors by latent class, workers with COPD in KY, TN, NC, and WV BRFSS respondents, 2016 and 2017. Binge drinking defined as ≥5 drinks for men or ≥4 drinks for women on one occasion during the past 30 days. Heavy drinking is defined as men having >14 drinks per week and women having >7 drinks per week. Behaviors refer to the past 30 days.

Over one third (38.02%) of workers with COPD living in Central Appalachian U.S. states belonged to the highest risk class for the five health risk behaviors (Figure 3). Of these, 85.27% smoked every day, 79.66% were heavy drinkers, and 50.00% reported binge drinking in the past 30 days. Slightly more than one-third (35.37%) of workers with COPD made up an intermediate risk class: 48.67% were heavy drinkers, 28.83% used e-cigarettes, and 21.71% reported binge drinking. In the remaining 26.60% of workers with COPD in the low-risk class, 54.05% were heavy drinkers and 28.83% reported binge drinking.

### 3.5. Health Care Access Risk Classes

**Figure 4:**
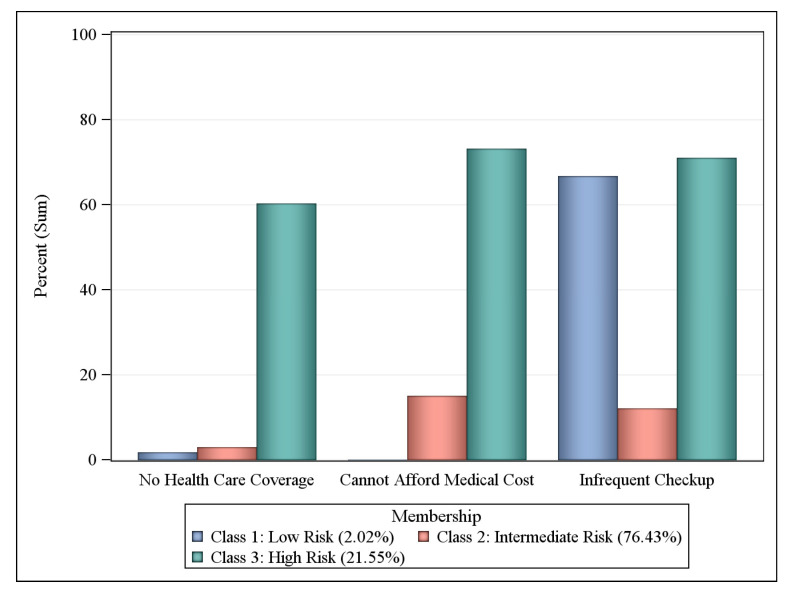
Probabilities of health care access by latent class, workers with COPD in KY, TN, NC, and WV BRFSS respondents, 2016 and 2017.

About 20 percent (21.55%) of workers with COPD living in Central Appalachian U.S. states belonged to the highest risk class for having difficulty accessing health care services (Figure 4). Of those at high risk, 72.96% could not afford medical costs, 72.44% had routine checkups more than one year ago, and 54.74% of patients reported having no health care plan. Due to the extremely low numbers of low-risk participants who reported they had no health insurance coverage and could not afford their medical costs, the low- and intermediate-risk groups were combined. In 78.45% of workers with COPD falling into the low-/intermediate-risk class, 78.64% had routine checkups over one year ago.

### 3.6. Influence of Risk Classes on HRQoL Outcomes

Table 2 presents the findings from the three logistic regression analyses assessing the association of latent classes with each of the three outcomes: (1) physical distress, (2) mental distress, and (3) activity limitation due to poor health. Workers with COPD at high risk for multiple comorbidities were over four (OR = 4.25, 95% CI = 2.03–8.89) times more likely to report frequent mental distress (*p* < 0.01), more than 2.50 (95% CI = 1.19–5.41) times more likely to report physical distress (*p* < 0.05) and about 4.15 (95% CI = 1.53–11.29) times more likely to report activity limitation due to poor health (*p* < 0.01) than respondents at low risk for comorbidities.

For health care access, latent risk classes were broken down into two categories (i.e., adequate access, limited access) due to small cells causing the proportion of respondents in the original low-risk class to be very small (1.57%) relative to the other two risk classes (i.e., intermediate and high). Following the reclassification into two classes (i.e., adequate access and limited access), multiple logistic regression analyses showed that those reporting limited access to healthcare were over two (OR = 2.22, 95% CI = 1.19, 4.11) times more likely to report limited activity due to poor health (*p* < 0.01) as compared to respondents reporting adequate health care access. However, respondents with limited access to healthcare were no more likely to report physical and mental distress as compared to respondents with adequate access to healthcare.

Workers with COPD at intermediate or high risk for substance use and abuse were not significantly more likely to report physical distress, mental distress, or limited usual activity due to poor health as compared to those at low risk.

### 3.7. GIS Results

**Figure 5:**
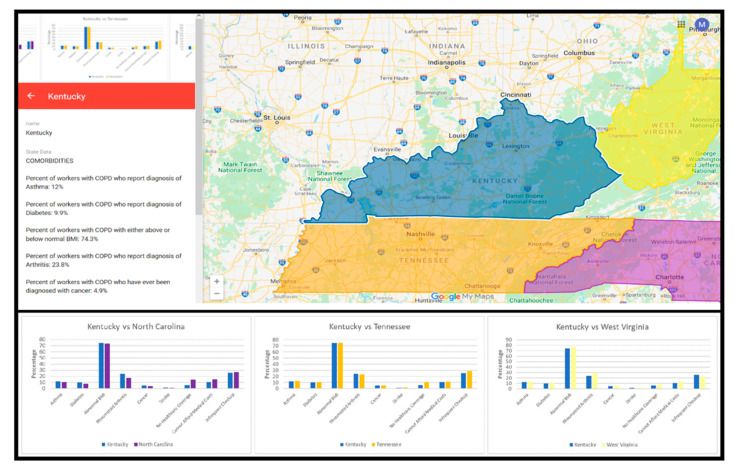
Google Map visualization depicting percentage of employed KY adults with COPD who reported comorbidities. Kentucky is selected on the Google Map. The percentage of BRFSS respondents with comorbid health conditions and health care access variables are displayed on the left side. Bar charts can be selected by clicking the upper right-hand corner to open pairwise state comparisons describing the percentage of KY respondents reporting each comorbidity as compared to respondents in each of the other three U.S. states (NC, TN, and WV).

The Google Map is an efficient visualization tool to disseminate our research results and supports a variety of interactions. In this project, we leveraged Google Map to visualize and compare state-level health data. Figure 5 shows state-level data for the statistically significant risk factors (i.e., comorbidities, health care access) associated with the outcome variables in the logistic regression analyses. U.S. State layer colors described the overall health risks of workers with COPD from each Central Appalachian state. As described in the Method section, the average percentage was computed by summing the average percentage of respondents reporting comorbid health condition variables (including asthma, diabetes, abnormal BMI, arthritis, cancer, and stroke) and inadequate healthcare access variable (including no access to health insurance coverage, difficulty affording medical costs, and time since last checkup). For each variable, we calculated the percentage of answers that indicated these statistically significant health risks were present. For example, abnormal BMI, a comorbidity, was classified into four categories: (1) underweight, (2) normal weight, (3) overweight, and (4) obesity. The percentage of abnormal BMI among workers with COPD in each Central Appalachian U.S. state was calculated and shown on the Google Map. Similarly, time since last checkup had four categories: (1) within the past year, (2) within the past two years, (3) within the past five years, and (4) more than five years. The percentage of workers with COPD reporting more than one year since their last checkup was calculated and displayed on the Google Map.

**Figure 6:**
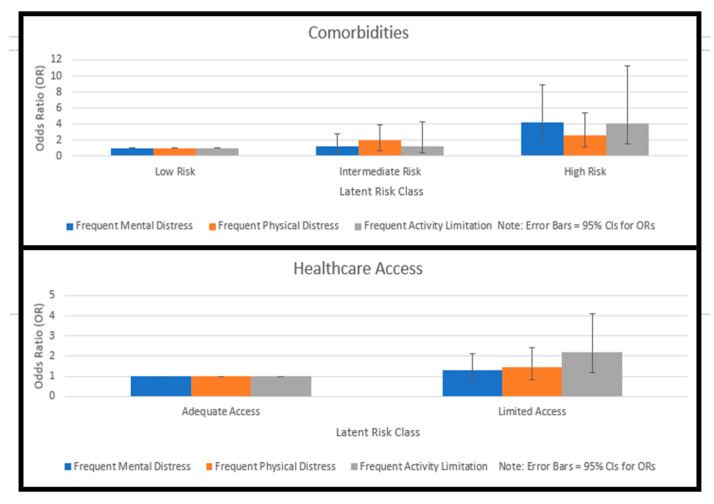
Odds ratio comparisons for latent risk classes of healthcare access and comorbidities as predictors of frequent physical distress, mental distress, and activity limitation among workers with COPD from U.S. states spanning the Central Appalachian region (i.e., KY, NC, TN, and WV). Layered bar graphs report odds ratios for each latent risk class of healthcare access and comorbidities from the logistic regression models predicting the three HRQoL outcomes.

The average respondent percentages represented the saturation value of each U.S. state, with higher percentages indicative of more serious health risk factors. The average state health risk percentages were the following: WV = 20.07%; KY = 19.72%; TN = 19.87%; and NC = 19.06%. The comparison of odds ratios associated with each health risk factor from each U.S. state were depicted in a layered bar chart accessible on the top left corner of the Google Maps interface. When the thumbnail image was clicked, the visualization of the odds ratios for the two statistically significant health risk indicator categories (i.e., comorbidities, health care access) was rendered (Figure 6). Results showed that the average worker in TN reported a comorbidity rate of 25.1%, which was higher than other Central Appalachian states (~21%). Similarly, the average percentage of workers without healthcare access in TN (17.1%) was higher than most other Central Appalachian states (~14%), except for NC (18.9%).

## 4. Discussion

This study examined variability in socio-ecological risk factors associated with poor HRQoL among adults with COPD who are employed and reside in Central Appalachia. Results demonstrate that approximately 20% of adult workers living with COPD in this predominantly rural region experience frequent physical and mental distress, with over 10% reporting limitations when engaging in their usual activities. Some socio-demographic factors increased the risk of physical and mental distress, but participants with comorbid conditions were at the greatest risk for experiencing poor HRQoL outcomes.

Younger workers with COPD were more likely to report mental distress as compared to those who are over 65 years old. This is consistent with study findings showing that young workers are at risk for mental health issues, with 12.5% of 21–25 years old and 17.2% of 18–20 years old reporting depression symptoms [39]. Younger workers between 18–30 years old perceived almost twice as much pressure in their lives compared to older workers [40]. Some factors affecting younger workers’ mental health issues may include work inexperience or under-preparedness, greater vulnerability to work pressures, financial concerns, and perceived lack of respect for them by coworkers [39,40].

Consistent with prior work, female workers with COPD were more likely to report mental distress as compared to their male counterparts [41]. Compared with men, women are more likely to be diagnosed with a mental health condition [42], including anxiety and depression [43], sleep disorders [44], and suicidal thoughts [43]. A recent survey of experts on women’s mental health in both low- and high-income countries indicated poor satisfaction with the availability of gender-sensitive services and identified the need for local and global action to address this disparity [45]. Employers must proactively address mental health issues among younger workers with COPD, especially those who identify as female, by providing psychological support and counseling for coping with mental distress.

Black/African American workers with COPD were less likely to report mental distress than their White counterparts. This finding is consistent with studies showing that once race-related stress is adjusted, mental stress affects white adults more adversely than racial/ethnic minorities [46,47]. Suggested reasons for such finding include: (1) better recovery among African Americans due to greater emotional flexibility in responding to stress [47,48]; and (2) greater access to helpful coping resources (e.g., religious involvement) among African Americans [46,47]. Further investigation on the factors associated with such racial differences in mental health is important for improving mental resilience among workers with COPD.

The relationship between socioeconomic status and COPD has been widely studied, generally supporting that low levels of income and education are associated with poor lung function and shortness of breath, among other health outcomes [49]. People with lower education generally reported a higher prevalence of mental distress [50] and mental disorders [51] compared to those with higher education. Consistently, workers with COPD who had a higher education (i.e., ≥4 years of college) were less likely to report mental distress than those without a high school diploma. Similarly, in the current study, workers with COPD who reported higher annual earnings (i.e., USD >35,000) were less likely to report mental and physical distress compared to those with lower income. Beyond SES, several studies have found contradicting outcomes to suggest that between-country differences, cohort differences, historical shifts and job–education mismatches may affect the association between education and mental health [52,53,54,55,56]. Future public health advocacy efforts should aim to increase the availability and accessibility of health promotion and worksite wellness support, with a focus on mental healthcare, for low-income workers in Central Appalachia. This includes challenging current health insurance reimbursement protocols using data that reinforces the association between low socioeconomic status and limited healthcare access, which both can contribute negatively to HRQoL of working adults with COPD in this underserved geographic region.

Three socio-ecological levels were considered as risk indicators, including the presence of comorbidities, engagement in substance use, and experiencing limited healthcare access. Nearly half (42.69%) of the participants were considered in the highest-risk class due to their daily smoking behaviors and heavy alcohol use. Similarly, nearly half (47.68%) of the sample was in the intermediate risk category, which most frequently included diagnoses of arthritis and obesity. Adults with COPD also commonly experience comorbid conditions, which makes it difficult for clinicians to recognize and coordinate care protocols for these patients. Finally, and most surprisingly, only about 18% of the sample was in the high-risk category for having difficulty accessing healthcare services due to either high medical costs, no access to a healthcare plan, and delayed treatment seeking.

Workers with COPD at intermediate or high risk for substance use and abuse were no more likely to report mental or physical distress and limited activity due to poor health compared to those at low risk. This result is supported by Pascal et al. [57] who concluded that mental conditions, specifically anxiety, depression and panic disorders, were constant characteristics of COPD patients, regardless of their current tobacco use. However, consistent with prior research [27], workers with COPD who were at high risk for multiple comorbidities were more likely to report physical distress, mental distress, and activity limitation due to poor health as compared to those at low risk for comorbidities. This finding in Central Appalachian workers with COPD is consistent with a study using national BRFSS data from the general COPD population, which found that those at risk for multiple comorbidities were more likely to report frequent physical and mental distress than the low-risk group [27]. Workers with lower incomes may be involved in more hazardous work environments where exposure to excess dusts, gases, and vapors is high. This may exacerbate COPD and its comorbidities, resulting in higher physical distress.

Moreover, our findings support that vulnerable workers with comorbidities of COPD must be targeted by workplace health programs in Central Appalachia, especially considering their susceptibility to both poor physical and mental health outcomes. Central Appalachian worksites should devote more resources towards helping their high-risk employees with COPD manage comorbidities such as asthma, arthritis, and unhealthy body weight. The presence of comorbidities can present challenges in terms of effectively self-managing COPD, primarily because standardized approaches and trials do not always account for the presence of interrelated conditions that often cause similar symptoms [58]. Workplace health promotion programs that target health behaviors important for general chronic disease management should adequately address fundamental topics, such as nutrition and physical activity, to positively impact the HRQoL of Central Appalachian employees with COPD.

Workers with COPD who reported less access to health care were more likely to report limited activity due to poor health as compared to those with adequate health care access. It is possible that the factors limiting access to health care may be the same ones that preclude workers with COPD from engaging in their regular activities, such as financial capability, lack of transportation and lack of a caretaker. For example, not having a vehicle or a driver available can be the same reason why a worker with COPD is not able to go to a health care facility and why he/she cannot travel (e.g., to the grocery). Moreover, limited access to health care also could have potentially caused or, at least, contributed, directly or indirectly, to poor health resulting in limited usual activities.

A few studies on HRQoL of employed adults with COPD in the U.S. that involved national data have been published [14,59], but there is none that involved a specific U.S. region outside of Central Appalachia. Thus, results in this study cannot be compared to other U.S. geographical regions to determine similarities and/or differences. Further investigation on the risk factors affecting HRQoL of workers with COPD in other U.S. regions is warranted.

The Google Map developed using state-level BRFSS data showed comparisons of statistically significant risk factor odds ratios for the comorbidity and healthcare access domains. State level comparisons using the Google map showed that the workers without healthcare access may also have elevated comorbidity rates. Based on the GIS analysis, it may be that NC has a lower percentage of workers with COPD who lack healthcare access and lower comorbidity rates than TN, because NC has instituted beneficial policies [60] that have improved air quality control and reduced death rates associated with chronic lung diseases [61]. Future research should explore how Central Appalachian environmental and occupational policies impact the HRQoL of workers with COPD. Such analyses should consider county-level data from U.S. states to create Google Maps that enable local comparisons within the Central Appalachian region.

This study is not without limitations. The secondary analysis included multiple years of national-level population data from the BRFSS, which are collected and managed by the Centers for Disease Control and Prevention. Given the nature of this secondary data analysis, we were limited in the number and content of items. For example, future research studying the association between risk factors and the HRQoL of workers living with COPD should consider incorporating more items related to other socio-ecological levels above and beyond the individual and health system (e.g., interpersonal relationships, community/social ties). Although the cross-sectional nature of data and the analyses of association are traditionally considered a limitation, the hypothesized associations were guided by a strong history of empirical literature and theoretical underpinnings supporting the causative relationship between risk indicators and outcomes.

## 5. Conclusions

Our analysis of 2016 and 2017 BRFSS data showed that about 1 in 5 workers with COPD in Central Appalachia reported frequent physical or mental distress, and nearly half were in the high-risk class for substance use and intermediate-risk class for comorbid conditions. While workers in the high-risk substance use and abuse class did not report poorer HRQoL than those at low risk, high-risk workers with comorbid conditions did report more frequent physical and mental distress as well as greater activity limitation due to poor health when compared to low-risk workers. These findings have important practice implications for informing policies and interventions that enable employed adults with COPD to retain productive employment while mitigating the effects of comorbidities that increase risk for poor HRQoL outcomes. To ensure that this knowledge is translated into meaningful practices that improve employee health outcomes, there is a need to effectively communicate these findings to communities and regional stakeholders (i.e., decision-makers such as healthcare agencies, occupational safety organizations and employment unions). Subsequent research should seek to understand more about how changes in worksite health promotion policies and interventions may help to reduce health disparities that disproportionately impact employees with COPD living in Central Appalachian states.

## Figures and Tables

**Table 1:** Prevalence of frequent physical distress, mental distress, and activity limitation by demographic characteristics, employed adults with COPD, Behavioral Risk Factor Surveillance System, KY, NC, WV, and TN, 2016–2017 ^1^.

Demographic Characteristic	No of Employed Adults with COPD ^1^, *n* = 1326 (% ^2^)	Frequent Mental Distress, % (95% CI) ^2^ *n* = 261 (19.68%)	Frequent Physical Distress, % (95% CI) ^2^ *n* = 278 (20.97%)	Frequent Usual Activity Limitation, % (95% CI) ^2^ *n* = 148 (11.16%)
*Age*				
18–44 years	379 (28.6)	42.9 (36.9, 48.9)	29.1 (23.8, 34.5)	28.4 (21.1, 35.7)
45–64 years	736 (55.5)	49.8 (43.7, 55.9)	57.2 (51.4, 63.0)	58.1 (50.1, 66.1)
≥65 years	211 (15.9)	7.3 (4.1, 10.4)	13.7 (9.6, 17.7)	13.5 (8.0, 19.0)
*Sex*				
Male	566 (42.7)	33.3 (27.6, 39.1)	39.6 (33.8, 45.3)	37.2 (29.4, 45.0)
Female	760 (57.3)	66.7 (60.9, 70.4)	60.4 (54.7, 66.2)	62.8 (55.0, 70.6)
*Race*				
White	1155 (87.1)	89.9 (86.2, 93.6)	87.3 (83.4, 91.2)	89.8 (84.9, 94.7)
Black or African American	106 (8.0)	5.4 (2.7, 8.2)	9.1 (5.7, 12.4)	4.8 (1.3, 8.2)
American Indian or Alaskan Native	53 (4.0)	4.7 (2.1, 7.2)	3.6 (1.4, 5.8)	5.4 (1.8, 9.1)
*Hispanic*				
Yes	30 (2.3)	3.1 (1.0, 5.2)	2.2 (0.4, 3.9)	2.7 (0.1, 5.3)
No	1290 (97.3)	96.9 (94.8, 99.0)	97.8 (96.1, 99.6)	97.3 (94.7, 99.9)
*Education*				
<High school degree	148 (11.2)	13.8 (9.6, 18.0)	13.0 (9.0, 16.7)	18.2 (12.0, 24.5)
Grade 12 or GED	524 (39.5)	40.6 (34.6, 46.6)	37.9 (32.2, 43.6)	32.4 (24.9, 40.0)
College 1–3 years	392 (29.6)	33.7 (28.0, 39.5)	35.7 (30.1, 41.4)	37.8 (30.0, 45.7)
College ≥4 years	258 (19.5)	11.9 (7.9, 15.8)	13.4 (9.3, 17.4)	11.5 (6.3, 16.6)
*Annual Income*				
USD <20,000	232 (17.5)	30.9 (24.9, 36.8)	30.0 (24.2, 35.8)	32.0 (23.8, 40.2)
USD 20,000–34,999	316 (23.8)	31.3 (25.3, 37.3)	27.9 (22.2, 33.6)	29.6 (21.6, 37.6)
USD 35,000–49,999	211 (15.9)	14.3 (9.8, 18.9)	16.7 (11.9, 21.4)	16.00 (9.6, 22.4)
USD 50,000–74,999	184 (13.9)	12.2 (7.9, 16.4)	12.9 (8.7, 17.2)	10.4 (5.0, 15.8)
USD ≥75,000	213 (16.1)	11.3 (7.2, 15.4)	12.5 (8.3, 16.7)	12.0 (6.3, 17.7)
*Marital Status*				
Married	629 (47.4)	40.4 (34.4, 46.4)	43.8 (37.8, 49.7)	41.8 (33.8, 49.8)
Divorced	292 (22.0)	25.0 (19.7, 30.0)	25.7 (20.6, 30.9)	23.3 (16.4, 30.2)
Widowed	126 (9.5)	8.5 (5.1, 11.8)	9.8 (6.3, 13.3)	16.4 (10.4, 22.5)
Separated	42 (3.2)	2.7 (0.7, 4.7)	3.3 (1.2, 5.4)	2.7 (0.1, 5.4)
Never married	187 (14.1)	17.3 (12.7, 21.9)	14.1 (10.0, 18.2)	12.3 (7.0, 17.7)
Unmarried couple	45 (3.4)	6.2 (3.2, 9.1)	3.3 (1.2, 5.4)	3.4 (0.5, 6.4)

^1^ BRFSS respondent inclusion criteria was answer of “Yes” for the question if ever told of COPD, emphysema, or chronic bronchitis diagnosis by health care provider and indicated being “employed for wages” or “self-employed” at the time of their interview. ^2^ Percentages reflect proportion of respondents experiencing distress within each demographic category subgroup. Some categories do not add to the total because of missing responses.

**Table 2:** Odds ratios (ORs) of frequent physical distress, mental distress, and activity limitation by individual and healthcare system risk domains, employed adults reporting diagnosis of COPD in KY, NC, WV, and TN, BRFSS, 2016–2017 ^1^.

Health Risk Domain	Frequent Mental Distress		Frequent Physical Distress		Frequent Activity Limitation	
	OR	95% CI	OR	95% CI	OR	95% CI
*Substance Use and Abuse*						
Low Risk (*n* = 300)	1.00 ^2^	ref	1.00 ^2^	ref	1.00 ^2^	ref
Intermediate Risk (*n* = 347)	0.90	0.42, 1.94	1.02	0.53, 1.95	0.71	0.29, 1.75
High Risk (*n* = 482)	1.23	0.67, 2.29	1.35	0.77, 2.37	0.69	0.33, 1.44
*Comorbidities*						
Low Risk (*n* = 316)	1.00 ^2^	ref	1.00 ^2^	ref	1.00 ^2^	ref
Intermediate Risk (*n* = 545)	1.21	0.53, 2,74	1.69	0.73, 3.90	1.28	0.39, 4.23
High Risk (*n* = 282)	4.25 **	2.03, 8.89	2.54 *	1.19, 5.41	4.15 **	1.53, 11.29
*Healthcare Access*						
Adequate Access (*n* = 1093)	1.00 ^2^	ref	1.00 ^2^	ref	1.00 ^2^	ref
Limited Access (*n* = 233)	1.28	0.77, 2.11	1.43	0.84, 2.43	2.22 **	1.19, 4.11

Notes: * *p* < 0.05; ** *p* < 0.01. ^1^ Analyses were adjusted for age, sex, race/ethnicity, education, marital status, annual income, and all other latent variables in Table 2. Frequent physical or mental distress was defined as reporting 14 or more physically or mentally unhealthy days in the last 30 days. ^2^ Reference values. Abbreviation: ref, reference category.
